# No evidence for a difference in lateralization and distinctiveness level of transcranial magnetic stimulation-derived cortical motor representations over the adult lifespan

**DOI:** 10.3389/fnagi.2022.971858

**Published:** 2022-10-13

**Authors:** Melina Hehl, Stephan P. Swinnen, Shanti Van Malderen, Koen Cuypers

**Affiliations:** ^1^Movement Control & Neuroplasticity Research Group, Department of Movement Sciences, Group Biomedical Sciences, KU Leuven, Heverlee, Belgium; ^2^Neuroplasticity and Movement Control Research Group, Rehabilitation Research Institute (REVAL), Hasselt University, Diepenbeek, Belgium; ^3^Leuven Brain Institute (LBI), KU Leuven, Leuven, Belgium

**Keywords:** cortical motor representation, aging, lateralization, distinctiveness, dedifferentiation, transcrancial magnetic stimulation (TMS)

## Abstract

This study aimed to investigate the presence and patterns of age-related differences in TMS-based measures of lateralization and distinctiveness of the cortical motor representations of two different hand muscles. In a sample of seventy-three right-handed healthy participants over the adult lifespan, the first dorsal interosseus (FDI) and abductor digiti minimi (ADM) cortical motor representations of both hemispheres were acquired using transcranial magnetic stimulation (TMS). In addition, dexterity and maximum force levels were measured. Lateralization quotients were calculated for homolog behavioral and TMS measures, whereas the distinctiveness between the FDI and ADM representation within one hemisphere was quantified by the center of gravity (CoG) distance and cosine similarity. The presence and patterns of age-related changes were examined using linear, polynomial, and piecewise linear regression. No age-related differences could be identified for the lateralization quotient of behavior or cortical motor representations of both intrinsic hand muscles. Furthermore, no evidence for a change in the distinctiveness of the FDI and ADM representation with advancing age was found. In conclusion this work showed that lateralization and distinctiveness of cortical motor representations, as determined by means of TMS-based measures, remain stable over the adult lifespan.

## Introduction

With aging, the central nervous system (CNS) undergoes deterioration that is associated with a decline in motor function (Borzuola et al., 2020). Furthermore, older as compared to younger adults show a generally lower performance level (Voelcker-Rehage, 2008) as demonstrated by slower reaction times (Seidler et al., 2010; Hermans et al., 2018, 2019), higher movement variability (Bedard et al., 2002; Wu and Hallett, 2005; Seidler et al., 2010), and impaired coordination (Greene and Williams, 1996; Swinnen et al., 1998; Serrien et al., 2000; Heuninckx et al., 2004). Imaging studies suggest that these changes in motor function are accompanied by age-related alterations in the structure (e.g., Barrick et al., 2010) and function of brain areas (e.g., King et al., 2018; Monteiro et al., 2019) during rest and task execution.

On the functional level, older subjects generally need to recruit more brain activity as compared to younger adults to successfully perform the same motor task (Heuninckx et al., 2008; Goble et al., 2010; Van Impe et al., 2011). This can relate to both, a more symmetric (less lateralized) activation (Sailer et al., 2000; Mattay et al., 2002; Ward and Frackowiak, 2003; Naccarato et al., 2006; Langan et al., 2010) and/or a general dedifferentiation of recruitment patterns, where additional brain areas within or across hemispheres are recruited in older as compared to young adults (e.g., Mattay et al., 2002; Seidler et al., 2010). Supporting this notion, older adults show an increased recruitment of motor (Sailer et al., 2000; Mattay et al., 2002; Ward and Frackowiak, 2003) and premotor areas (Calautti et al., 2001; Mattay et al., 2002; Ward and Frackowiak, 2003) during the execution of simple unimanual motor tasks such as a hand grip (Ward and Frackowiak, 2003), button press (Mattay et al., 2002), or finger tapping (Sailer et al., 2000; Calautti et al., 2001). In addition to the studies above, which were focused on task-related brain activation, functional connectivity research revealed a reduced segregation and modulation of functional networks in older adults performing a complex bimanual motor tasks, which was associated with a decreased motor performance (King et al., 2018; Monteiro et al., 2020).

As compared to functional brain imaging research, the aging effect on the symmetry and distinctiveness of the primary motor cortex’ (M1) excitability patterns have to date received little attention. Moreover, existing transcranial magnetic stimulation (TMS) studies reported contradictory findings about the evolution of cortical motor representations (also referred to as motor maps) with increasing age for muscles involved in dexterous activities, such as the first dorsal interosseus (FDI) that is involved in performing the precision grip (Bernard and Seidler, 2012; Coppi et al., 2014; Hehl et al., 2020). Bernard and Seidler (2012) found a larger cortical motor representation for the FDI with advancing age, irrespective of the investigated hemisphere, which they interpreted as a sign of dedifferentiation in the aging brain. In contrast, other studies found no spatial alterations of the FDI representation in the dominant M1 with age (Hehl et al., 2020) or even a decline in the extent of the dominant, but not non-dominant, abductor pollicis brevis (APB) representation (Coppi et al., 2014). For the abductor digiti minimi (ADM), a hand muscle less involved in fine motor tasks, no age-related differences in the spatial extent of the M1 representation have been reported for the dominant (Hehl et al., 2020) or both hemispheres (Coppi et al., 2014). Hence, a differential effect of aging on the excitability patterns of different hand muscle motor representations with increasing age can be assumed.

This distinct aging effect on different hand muscle representations in M1 (e.g., FDI and ADM, respectively, contributing to precision and power grip) might be linked to changes in TMS-based measures of lateralization (i.e., the balance between homolog representations of both hemispheres) and/or distinctiveness (here defined as the spatial delineation and degree of overlap of different hand muscle representations within a hemisphere) in older as compared to younger adults. Therefore, we explored the lifespan patterns in the lateralization and distinctiveness of two intrinsic hand muscle representations in the resting M1. We hypothesized (1) a difference in the lateralization of motor skill (Przybyla et al., 2011; Graziadio et al., 2015) and cortical motor representations (Sailer et al., 2000; Calautti et al., 2001; Mattay et al., 2002; Ward and Frackowiak, 2003; Naccarato et al., 2006; Langan et al., 2010) with advancing age; and (2) a difference in the level of distinctiveness of the within-hemisphere cortical motor representations of the two intrinsic hand muscles FDI and ADM with advancing age (Sailer et al., 2000; Calautti et al., 2001; Mattay et al., 2002; Ward and Frackowiak, 2003).

## Materials and methods

### Participants

Seventy-three healthy right-handed participants over the full adult lifespan [aged 18–81 years, mean age ± SD: 50.44 ± 17.78, 35 female, Edinburgh Handedness Inventory (Oldfield, 1971) mean lateralization quotient (LQ) ± SD: 92.57 ± 13.96] were recruited on community and university level in Flanders, Belgium, and included in this cross-sectional study [right-handed subsample of the study cohort described in Hehl et al. (2020) as there is evidence for differences in lateralization for left- as compared to right-handed individuals (Civardi et al., 2000; Reid and Serrien, 2014)]. It should be noted that although the data reported in the current work partially overlaps with a previous report (Hehl et al., 2020), the current focus is on age-related changes in TMS-based measures of lateralization and distinctiveness (including maps of both hemispheres) instead of identifying the relationship between sensorimotor function and motor representations in the dominant hemisphere only. Prior to inclusion, subjects were screened and excluded from participation if they reported any CNS or psychiatric disorders, neuroactive medication intake, history of brain injury or surgery, or contraindications for TMS (Wassermann, 1998). At the start of the study, participants completed the Montreal Cognitive Assessment (MoCA) (Nasreddine et al., 2005) (mean score ± SD: 28.23 ± 1.59, range 24–30) and were excluded if they scored below 24 points (Rossetti et al., 2011; Ciesielska et al., 2016). Furthermore, the self-reported Baecke questionnaire (Baecke et al., 1982; Voorrips et al., 1991) (final scores can range from 3 to 15 representing lowest and highest physical activity, respectively; mean score ± SD: 8.30 ± 1.15) was administered to evaluate the participants’ habitual physical activity. Participant characteristics are summarized in Table 1. The study protocol was approved by the local ethics committee (Ethics Committee Research UZ/KU Leuven; reference S62231) and participants gave full written informed consent prior to study participation, complying with the latest amendment of the Declaration of Helsinki (World Medical Association, 2013).

**TABLE 1:** Participant characteristics.

Category	Total	18–30 years	31–40 years	41–50 years	51–60 years	61–70 years	71–81 years
Participants	73 (100%)	12 (16.4%)	14 (19.2%)	12 (16.4%)	10 (13.7%)	13 (17.8%)	12 (16.4%)
Age (years)	50.44 ± 17.78	24.75 ± 4.07	35.64 ± 3.05	47.00 ± 2.63	55.50 ± 2.51	66.31 ± 3.01	75.42 ± 2.64
Female	35 (47.9%)	6 (50.0%)	6 (42.9%)	7 (53.8%)	5 (50.0%)	7 (53.8%)	4 (33.3%)
rMT LH (% MSO)	35.56 ± 6.78	38.42 ± 6.99	36.36 ± 7.27	35.42 ± 8.06	30.60 ± 11.72	34.62 ± 7.25	34.08 ± 5.48
rMT RH (% MSO)	36.71 ± 7.71	38.17 ± 7.06	37.00 ± 8.00	38.25 ± 9.72	35.40 ± 5.58	36.54 ± 8.69	34.67 ± 7.05
EHI LQ (%)	92.57 ± 13.96	80.05 ± 14.96	94.07 ± 8.66	95.70 ± 10.34	96.64 ± 7.11	98.60 ± 5.04	90.30 ± 23.22
MoCA	28.23 ± 1.59	29.00 ± 1.48	28.29 ± 1.54	28.33 ± 1.23	28.20 ± 1.87	28.38 ± 1.50	27.17 ± 1.70
Baecke	8.30 ± 1.15	8.50 ± 0.96	8.29 ± 1.71	8.57 ± 0.97	8.09 ± 1.05	8.48 ± 1.25	7.80 ± 0.54

In addition to the pooled group data (Total), data for subdivisions of six age groups are reported for illustrative purposes. Data are displayed as total number (and percentage) or as mean ± standard deviation.

Baecke, Baecke Questionnaire of Habitual Physical Activity; EHI LQ, lateralization quotient assessed by Edinburgh Handedness Inventory; LH, left hemisphere; MoCA, Montreal Cognitive Assessment; MSO, maximal stimulator output; RH, right hemisphere; rMT, resting motor threshold.

### Transcranial magnetic stimulation and electromyographic recordings

Starting with the right or left hemisphere in a semi-randomized order, biphasic single-pulse TMS pulses were delivered to the M1 of each hemisphere using a 70 mm figure-of-eight coil (MC-B70, outer coil winding diameter 2 × 97 mm, 150° angled) connected to a MagPro X100 stimulator (MagVenture A/S, Farum, Denmark). In short, participants were seated in a chair with their forearms pronated. Contralateral to the stimulated hemisphere, EMG signals [filtered for 50/60 Hz noise (Humbug, Quest Scientific, North Vancouver, Canada), gain = 1000, 20–2000 Hz bandpass filter, sampling rate = 5000 Hz] of the FDI and ADM muscle were collected using surface Ag-electrodes (Bagnoli™ DE-2.1 EMG Sensors and Bagnoli-4 EMG System, DELSYS Inc., Boston, MA, USA) fixed to the belly of each muscle with a reference electrode at the dorsal side of the wrist. Participants were comfortably seated to ensure the EMG signal root mean square (RMS) was below 5 μV between successive TMS pulses, which was continuously monitored. Accurate coil placement and coil orientation [handle pointing backward and 45° away from the midline, delivering biphasic stimuli to elicit a second phase posterior-to-anterior directed current in the cortex, coil center positioned tangentially to the scalp (Brasil-Neto et al., 1992; Mills et al., 1992)] were ensured using optically tracked neuronavigation (Brainsight^®^2, Rogue Research Inc, Montreal, Quebec, Canada).

For each hemisphere, a standardized TMS protocol was performed. Firstly, the vertex was determined (Klem et al., 1999). This landmark served as the center of a 1 cm-spaced 19 × 19 grid, which was projected with Brainsight^®^. Using this grid, the FDI hotspot, i.e., the scalp location with the strongest and most consistent MEP in the FDI averaged over 5 consecutive TMS pulses was determined. At the hotspot, the lowest stimulation intensity resulting in ≥5/10 MEPs with a peak-to-peak amplitude >50 μV in the relaxed FDI was defined as resting motor threshold (rMT) (Rossini et al., 2015). Then, the cortical motor representations of the FDI and ADM were mapped using a 1 cm-spaced 11 × 11 grid centered around the hotspot. Per target point, 8 consecutive pulses (inter-trial interval: 3 s ± 20%) were administered at an intensity of 115% rMT (Malcolm et al., 2006; Coppi et al., 2014), starting at the hotspot and proceeding spirally clockwise until all active points, i.e., points with ≥4/8 MEPs of ≥100 μV peak-to-peak amplitude in at least one of the target muscles, were surrounded by inactive points. If at least in one of the 8 pulses an EMG background activity of ≥20 μV (see Cuypers et al., 2020) was present in the FDI or ADM during the 50 ms preceding the TMS pulse (Cuypers et al., 2020), the measurement of that spot was immediately repeated. During the TMS mapping, all participants were blinded from EMG signals and watched a slideshow of landscape pictures to promote a stable level of alertness. After finalizing the mapping procedure for the first hemisphere, a 10-min break was provided before repeating the procedure for the second hemisphere. The entire TMS experiment lasted approximately 2 h (preparation included). For a more detailed explanation and visualization of the TMS data acquisition procedures, please see Hehl et al. (2020).

### Sensorimotor performance

Sensorimotor performance was assessed after the TMS protocol in order to not interfere with the TMS data acquisition. To measure grip and palmar pinch force, a hydraulic hand dynamometer (Model SH5001, Saehan Corporation, Masan, Korea) and a pinch force sensor (LCM302-200N, Omega Engineering Inc, Norwalk, CT, USA) were used, respectively. Participants were standing upright with the elbow flexed at 90°. For each side and test, the maximally generated force out of three verbally encouraged trials was recorded.

As a measure of manual dexterity, the number of pegs placed within a 30-s period on the unimanual subtest of the Purdue Pegboard Test (PPT, Model 32020, Lafayette Instrument Company Inc, IN, USA) was recorded for each hand.

### Electromyography data processing

Electromyography (EMG) data was processed using MATLAB^®^ (R2018b, The MathWorks Inc, Natick, MA, USA). Firstly, the cortical motor representation area (AREA) was calculated as the area of a polygon expanding over all active points, cortical motor representation volume (VOL) was calculated as the sum of the mean MEP peak-to-peak amplitudes of all active points, and the largest averaged MEP (MAXMEP) among all active points of the cortical motor representation was determined [see Hehl et al. (2020) for visualization of these parameters]. Secondly, the center of gravity (CoG) of the cortical motor representation of each muscle was calculated, using the formula: *CoG* = [*a*_*i*_*x*_*i*_/*a*_*i*_,*a*_*i*_*y*_*i*_/*a*_*i*_], for grid coordinates (*x*_*i*_,*y*_*i*_) and amplitudes *a_i_* (Wassermann et al., 1992; Meesen et al., 2011).

Lateralization of sensorimotor and neurophysiological measures was expressed using the lateralization quotient *LQ* = ([*X*_*DOM*_−*X*_*nDOM*_]/[*X*_*DOM*_ + *X*_*nDOM*_])×100 with values ranging between +100 and −100, indicating a strong asymmetry toward the dominant or non-dominant side/hemisphere, respectively (Binder et al., 1996; Houle and Tremblay, 2020). Values around 0 indicated symmetry.

**FIGURE 1:**
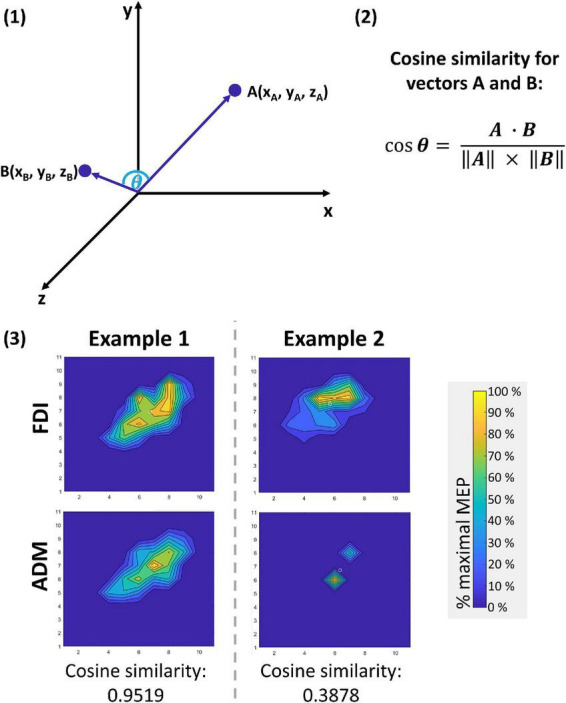
Cosine similarity approach to compare vectors with n dimensions. With this approach, the elements of two vectors with equal dimensionality n can be compared. In the three-dimensional example given for illustrative purposes in panel **(1)** below, the vectors A and B each contain three elements (*n* = 3) that define their parameter values for the characteristics x, y, and z. Assuming that characteristics x, y, and z can only adopt values ≥0 (as given in the present use case), the angle θ between the two vectors can adopt values between 0° (perfectly similar vectors that might only vary in length) and 90° (no similarity between the two vectors). By calculating the cosine of using the formula given in panel **(2)**, the outcome ranges between 0 (no similarity) and 1 (perfect similarity). This approach can likewise be applied to a vector with *n* = 121 dimensions, as has been done for comparing cortical motor representations for the first dorsal interosseus (FDI) and the abductor digiti minimi (ADM) muscle in this study. In this use case, the dimensionality of the vectors for FDI and ADM each equal the number of the 11 × 11 grid points (*n* = 121) and the mean motor evoked potential (MEP) amplitudes of each point are the elements of the vectors for FDI and ADM. Two examples are given in panel **(3)**.

To measure the distinctiveness of the cortical motor representations of FDI and ADM, the distance between CoG locations and the 3-dimensional topography of these motor representations were investigated. To do so, firstly the Euclidean distance between the CoG locations of FDI and ADM within one hemisphere was calculated: $d\,i\,s\,t\,\left(\left(x_{F\,D\,I},y_{F\,D\,I}\right),\left(x_{A\,D\,M},y_{A\,D\,M}\right)\right)=\sqrt{{\left(x_{F\,D\,I}-x_{A\,D\,M}\right)}^{2}+{\left(y_{F\,D\,I}-y_{A\,D\,M}\right)}^{2}}$. As a second measure of similarity, the cosine similarity (for details see Figure 1) was computed to quantify the (dis)similarity of the FDI and ADM motor representation volumes, thus referring to their 3-dimensional topography (Mitchell et al., 2008; Bruffaerts et al., 2013; Neyens et al., 2017). Individual muscle representations for both FDI and ADM were expressed as a vector with 121 dimensions (equivalent to the number of the 11 × 11 = 121 grid points) with the mean MEP amplitude of each grid point as the 121 elements of the vector. The calculated cosine similarity between the FDI and the ADM vector for each participant and hemisphere could range between 0 (no similarity), and 1 (perfect similarity). This method was favored as compared to the Jaccard index (McGregor et al., 2012; Grab et al., 2018), as the latter only takes into account the location but neither the size, nor the magnitude of excitability-based motor maps.

### Statistical analysis

R [Version 3.5.1, (R Core Team, 2018); *locfit* package version 1.5–9.1 (Loader, 2013); *ggplot2* package (Wickham, 2016) for visualizations] was used to perform statistical analyses on the TMS and performance data with α set to 0.05.

In order to identify differences in the pattern of TMS-derived motor map lateralization and distinctiveness across the lifespan, a standardized stepwise regression approach was applied [a visualization of this approach can be found in Hehl et al. (2020)]. Firstly, a locally weighted regression [local linear fit, tri-cube weight function, smoothness (alpha) = 0.5 (Cleveland, 1979); no robustness] was fitted to the data and visually inspected for its shape. Locations of breakpoints in the fit (minimum, maximum, sudden change in slope) were identified. Secondly, linear, polynomial (until 4*^th^* order), and piecewise linear regressions (one per breakpoint as identified by the locally weighted regression) were modeled. Thirdly, by determining the lowest Akaike Information Criterion (AIC) among the significant regression models, the best fitting regression was identified, while monitoring the significance of the slope parameter estimates and the R^2^_*adjusted*_ value. Lastly, it was verified whether the significant chosen model met the assumptions (homoscedasticity and normal distribution of the residuals) by visually inspecting the residuals’ normalized histogram, the normalized quantile-quantile (Q-Q) plot, and the Cook’s distance plot. If the model assumptions were not met, the influence of data transformation and/or removal of influential data points, as identified by the Cook’s distance plot, were analyzed to comply with the assumptions.

The described step-by-step approach has been favored over other, more commonly used approaches such as the comparison of different age groups or correlation analyses, as information gets lost with the categorization of continuous data. This might not only mask the actual relationship between two variables, but also compromises the statistical power with an increased risk of false positive results (Altman and Royston, 2006).

## Results

For the quotients and variables analyzed in this paper, each calculation required two separate measurement results (e.g., the LQ requires measurements of the dominant and non-dominant hemisphere). As both, intra- and interhemispheric comparisons were investigated, a single missing outcome led to more than one missing data point in the final variables. An overview of non-existent outcomes and the reasons for missing data are presented in Table 2. The resulting number of data points included in the regression analyses are summarized in Table 3.

**TABLE 2:** Overview of missing outcomes within the total sample of 73 participants and justification.

Measurement	Missing outcomes in total *n* = 73 (reason for missing)			
	
	Dominant side/hemisphere		Non-dominant side/hemisphere	
		
	FDI	ADM	FDI	ADM
**Behavioral tests** (PPT, GRIP FORCE, PINCH FORCE)	–	–	–	–
**rMT**	1* (high rMT)	n/a	–	n/a
**Cortical motor representation** (AREA, VOL, MAXMEP)	1* (high rMT)	1* (high rMT) 9^†††^ (no MEPs at 115% rMT of FDI)	–	8^†††^ (no MEPs at 115% rMT of FDI)

ADM, abductor digiti minimi; FDI, first dorsal interosseus; MAXMEP, maximal motor evoked potential; MEP, motor evoked potential; n/a, not applicable; PPT, Purdue Pegboard Test; rMT, resting motor threshold; VOL, volume. *Data points of the same participant that are missing for the same reason (e.g., a too high rMT prevented the data acquisition of cortical motor representations for both muscles). ^†††^In three of these participants, ADM motor representations were missing on both hemispheres.

**TABLE 3:** Number of included data points for each variable included in the analyses.

Variable	Number of included data points
**Age**	73
**Lateralization quotients**
• Behavioral tests:	73
− LQ_PPT, LQ_GRIP_FORCE, LQ_PINCH_FORCE
• rMT (LQ_RMT)	72
• Cortical motor representations of FDI/ADM:	72 FDI/58 ADM
−LQ_AREA_FDI/ADM
−LQ_VOL_FDI/ADM
−LQ_MAXMEP_FDI/ADM
**Similarity and COG Euclidean distance** Dominant/non-dominant hemisphere:	63 DOM/65 nDOM
− COGdist_DOM/nDOM
− CosSim_DOM/nDOM

ADM, abductor digiti minimi; DOM, dominant hemisphere; FDI, first dorsal interosseus; LQ, lateralization quotient; MAXMEP, maximal motor evoked potential; MEP, motor evoked potential; nDOM, non-dominant hemisphere; PPT, Purdue Pegboard Test; rMT, resting motor threshold; VOL, volume.

### Age-related differences in lateralization

**FIGURE 2:**
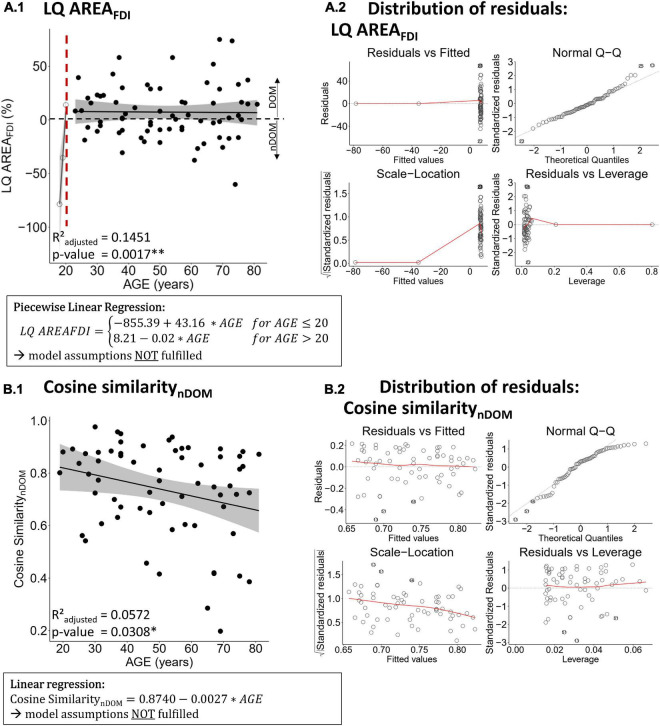
Best regression fits for the lateralization quotient of the first dorsal interosseus (FDI) motor area [LQ AREA_*FDI*_, panel **(A.1)**] and the cosine similarity between the FDI and abductor digiti minimi (ADM) representation of the non-dominant hemisphere [Cosine Similarity_*nDOM*_, panel **(B.1)**]. Panel **(A.2,B.2)** show the distribution of the residuals, respectively, for LQ AREA_*FDI*_ and Cosine Similarity_*nDOM*_ regressions. Estimates of significant regression models are stated in a rectangle below the plot and the regression is indicated in the plot by a solid line. Ribbons depict the 95% confidence interval of the fit. Significant p-values are indicated with asterisks (** *p* < 0.01; * *p* < 0.05) and printed in bold.

There was no evidence for a difference in the LQ of motor performance with increasing age (all *p* ≥ 0.4859, all *R*^2^_*adjusted*_ ≤ −0.0071). The LQ of the cortical motor representation area of the FDI was best modeled by a piecewise linear regression with a breakpoint at the age of 20 years (*p* = 0.0017, *R*^2^_*adjusted*_ = 0.1451; see Figure 2.A.1). This regression was a clear overfit and did not comply with the model assumptions (see Figure 2.A.2). All attempts to comply with the model assumptions and all other regression models were non-significant (all *p* > 0.05). All other regression models for age-related differences in the LQ of TMS measures were non-significant (all *p* ≥ 0.2452, all *R*^2^_*adjusted*_ ≤ 0.0153; see Table 4 for detailed information on all models).

**TABLE 4:** Point estimates, standard error (SE) and statistics for regression models for lateralization quotients [LQ = (R-L)/(R+L)] with AGE as independent variable.

Dependent variable (best fitting regression model)		Estimate:	SE:	t-value:	P(>|t|):	R^2^ adjusted:	F-statistic (DF NUM; DF DEN):	P(>F):
***LQ**_**PPT**∼**AGE***	Intercept	3.3843	2.4494	1.382	0.1710	−0.0114	*F* (1;71) = 0.1872	0.6666
(linear regression)	β1	−0.0198	0.0458	−0.433	0.6670			
***LQ**_**GRIP**∼**AGE***	Intercept	1.9257	2.1099	0.913	0.3640	−0.0071	*F* (1;71) = 0.4907	0.4859
(linear regression)	β1	−0.0277	0.0395	−0.700	0.4860			
***LQ**_**PINCH**∼**AGE***	Intercept	−2.6273	3.9616	−0.663	0.5090	−0.0138	*F* (1;71) = 0.01885	0.8912
(linear regression)	β1	−0.0102	0.0741	−0.137	0.8910			
***LQ**_**RMT**∼**AGE***	Intercept	4.8140	4.2261	1.139	0.2590	0.0050	*F* (2;69) = 1.177	0.3142
(piecewise linear regression,	β1	−0.1716	0.1123	−1.529	0.1310			
breakpoint: AGE = 47)	β2	0.2603	0.1785	1.458	0.149			
***LQ**_**AREA_FDI_**∼**AGE***	Intercept	−855.39	235.94	−3.625	**0.0005** ***	0.1451	*F* (2;69) = 7.026	0.0017**
(piecewise linear regression,	β1	43.16	11.89	3.630	**0.0005** ***			
Breakpoint: AGE = 20 ‡)	β2	−43.18	11.94	−3.616	**0.0006** ***			
***LQ**_**AREA_ADM_**∼**AGE***	Intercept	25.3917	20.8941	1.215	0.2295	0.0153	*F* (2;55) = 1.442	0.2452
(piecewise linear regression,	β1	−0.8691	0.5626	−1.545	0.1282			
Breakpoint: AGE = 46)	β2	1.4479	0.8544	1.695	0.0958			
***LQ**_**VOL_FDI_**∼**AGE***	Intercept	6.4518	17.0448	0.379	0.7060	−0.0141	*F* (1;70) = 0.01151	0.9149
(linear regression)	β1	−0.0342	0.3188	−0.107	0.915			
***LQ**_**VOL_ADM_**∼**AGE***	Intercept	−5.7519	17.9253	−0.321	0.7490	−0.0153	*F* (1;56) = 0.1422	0.7076
(linear regression)	β1	−0.1243	0.3295	−0.377	0.708			
***LQ**_**MAXMEP_FDI_**∼**AGE***	Intercept	−60.6510	51.5980	−1.175	0.244	0.0033	*F* (2;69) = 1.118	0.3329
(piecewise linear regression,	β1	2.2930	1.7680	1.297	0.199			
Breakpoint: AGE = 32)	β2	−2.7350	1.9600	−1.396	0.167			
***LQ**_**MAXMEP_ADM_**∼**AGE***	Intercept	2.3014	15.6585	0.147	0.884	−0.0098	*F* (1;56) = 0.4483	0.5059
(linear regression)	β1	−0.1927	0.2879	−0.670	0.506			

The following regression models were used: linear regression $\overset{\wedge }{\text{Y}}$ = β_0_ + β_1_*X*; quadratic regression $\overset{\wedge }{\text{Y}}$ = β_0_ + β_1_*X* + β_2_*X*^2^; piecewise linear regression $\overset{\wedge }{\text{Y}}$ = β_0_ + β_1_*X* + β_2_*X*^2^(*X* − *X_BP_*)×D with D = $\left\{ \begin{array}{l} 0\;for\;X\;\leq \;X_{BP}\\ 1\;for\;X\;>X_{BP} \end{array}\right.$ (BP = breakpoint). Significant p-values are indicated with asterisks (*** *p* < 0.001; ** *p* < 0.01) and printed in bold.

‡ = Not fulfilling model assumptions; all attempts to comply such as transformations and removal of influential data points lead to non-significant models.

ADM, abductor digiti minimi muscle; AREA, area of cortical motor representation; DEN, denominator; DF, degrees of freedom; FDI, first dorsal interosseus muscle; LQ, lateralization quotient; MAXMEP, maximal motor evoked potential of cortical motor representation; NUM, numerator; PPT, Purdue Pegboard Test unimanual subtest; SE, standard error; VOL, volume of cortical motor representation.

### Age-related differences in motor map distinctiveness

Only cosine similarity of the non-dominant hemisphere showed a significant relationship with age that was best modeled by a linear regression (*p* = 0.0308, *R*^2^_*adjusted*_ = 0.0572; see Figure 2.B.1) indicating less similarity between FDI and ADM of the non-dominant hemisphere with advancing age. However, this model did not fulfill the model assumptions (left-skewed distribution of residuals, see Figure 2.B.2) and all attempts to comply (data transformation including Box Cox transformation, analysis of influential data points) were not leading to a conformance with the model assumptions. As non-parametric alternative to a linear regression, a Spearman’s correlation was conducted and turned out to be non-significant (Spearman’s ρ = −0.2204; *p* = 0.0777). All other regressions for cosine similarity and CoG distance with age were non-significant (all *p* ≥ 0.2292, all *R*^2^_*adjusted*_ ≤ 0.0157; see Table 5 for detailed information on all models).

**TABLE 5:** Point estimates, standard error (SE) and statistics for regression models for similarity of first dorsal interosseus (FDI) and abductor digiti minimi (ADM) with AGE as independent variable.

Dependent variable (best fitting regression model)		Estimate:	SE:	t-value:	P(>|t|):	R^2^ adjusted:	F-statistic (DF NUM; DF DEN):	P(>F):
***COGdistance _DOM_**∼**AGE***	**Intercept**	0.4952	0.0892	5.553	<0.0001***	−0.0126	*F* (1;61) = 0.229	0.6340
(linear regression)	**β1**	−0.0008	0.0016	−0.478	0.6340			
***COGdistance _nDOM_**∼**AGE***	**Intercept**	0.3031	0.1302	2.328	0.0232*	0.0157	*F* (2;62) = 1.509	0.2292
(piecewise linear regression,	**β1**	0.0045	0.0029	1.569	0.1218			
breakpoint: AGE = 63)	**β2**	−0.0174	0.0103	−1.680	0.0980			
***CosSim _DOM_**∼**AGE***	**Intercept**	0.7678	0.0696	11.030	**<0.0001** ***	−0.0107	*F* (1;61) = 0.3412	0.5613
(linear regression)	**β1**	−0.0007	0.0013	−0.584	0.561			
***CosSim _nDOM_**∼**AGE***	**Intercept**	0.8740	0.0655	13.337	**<0.0001** ***	0.0572	*F* (1;63) = 4.881	0.0308*
(linear regression)	**β1**	−0.0027	0.0012	−2.209	**0.0308** *			

The following regression models were used: linear regression $\overset{\wedge }{\text{Y}}$ = β_0_ + β_1_*X*; quadratic regression $\overset{\wedge }{\text{Y}}$ = β_0_ + β_1_*X* + β_2_*X*^2^; piecewise linear regression $\overset{\wedge }{\text{Y}}$ = β_0_ + β_1_*X* + β_2_*X*^2^(*X* − *X_BP_*)×D with D = $\left\{ \begin{array}{l} 0\;for\;X\;\leq \;X_{BP}\\ 1\;for\;X\;>X_{BP} \end{array}\right.$ (BP = breakpoint). Significant p-values are indicated with asterisks (*** *p* < 0.001; * *p* < 0.05) and printed in bold.

COG, center of gravity; CosSim, cosine similarity; DEN, denominator; DF, degrees of freedom; DOM, dominant hemisphere; nDOM, non-dominant hemisphere; NUM, numerator; SE, standard error.

## Discussion

We explored the pattern in the lateralization and distinctiveness of two intrinsic hand muscle representations in the resting M1 over the lifespan, as assessed with single-pulse TMS. Firstly, based on the general aging literature, we hypothesized a difference in lateralization of cortical motor representations with advancing age. However, no significant age-related differences in the LQ of FDI and ADM motor representations were observed. The only exception was a significant regression of age on the FDI area lateralization, that did not comply with the model assumptions and can most likely be ascribed to an overfitted regression model. Secondly, we hypothesized a difference in the level of distinctiveness of the cortical motor representations of the two intrinsic hand muscles FDI and ADM with advancing age. While there were no age-related differences in similarity between the two muscle representations of the dominant hemisphere or their CoG distance, the non-dominant hemisphere showed a decreasing similarity between FDI and ADM representations, thus suggesting an increased distinctiveness with advancing age. However, this result must be interpreted with caution, as the assumptions for this regression model could not be met and non-parametric testing led to non-significant findings.

### Lateralization

In the present sample, no evidence for age-related differences in the lateralization of behavioral measures of force (hand grip force, pinch force) or dexterity (unimanual PPT performance) was identified. To date, literature on the aging effect of motor performance lateralization is discordant. For example, evidence suggests that younger adults show more performance asymmetry on a multidirectional reaching task than older adults (Przybyla et al., 2011) and have a higher intermanual skill transfer on a myoelectric-controlled interface learning task (Graziadio et al., 2015), which is in contrast to the current findings. In another study, Schaefer (2015) indicated that the performance asymmetry between the two hands on a simulated dressing or feeding task did not change with increasing age, which is in support of the present results. Therefore, the presence or absence of asymmetry changes in motor performance with increasing age might depend on the dexterity and cognitive demands of a particular motor task.

Regarding the neurophysiological aspect, previous literature pointed toward a decreased lateralization of brain activity with advancing age. More specifically, prior functional magnetic resonance imaging (fMRI) evidence suggests a more bilateral brain activity in older as compared to younger adults during the execution of various motor tasks such as hand grip (Ward and Frackowiak, 2003), thumb opposition (Naccarato et al., 2006), unimanual joystick (Langan et al., 2010) and button press tasks (Mattay et al., 2002), indicating a decrease in lateralization of motor-related brain activity. This is also supported by electro-encephalography (EEG) evidence, e.g., for a finger tapping task (Sailer et al., 2000; Rosjat et al., 2018). Potential underlying mechanisms could be the decline in white matter integrity (Barrick et al., 2010) and decrease in interhemispheric inhibition (Talelli et al., 2008a,b; Langan et al., 2010; Fling and Seidler, 2012) in the aging brain.

In contrast to the task-related fMRI and EEG studies discussed in the previous paragraph, the present work suggests no evidence for lateralization differences over the adult lifespan in the cortical TMS-derived motor maps of intrinsic hand muscles as acquired at rest. Although a study by McGregor et al. (2012) observed a good correspondence between task-related (contraction) fMRI maps and resting-state TMS maps, it could be argued that fMRI evidence from other, more complex task paradigms, differ more from TMS-derived motor maps at rest. Similar to task-related fMRI studies, resting-state fMRI network analysis supports the notion of brain dedifferentiation. More specifically, the overall connectivity within networks has been shown to decrease in older as compared to younger adults (Song et al., 2014; Vidal-Piñeiro et al., 2014; Staffaroni et al., 2018; Monteiro et al., 2019). In contrast, the connectivity between networks is increased in older as compared to younger adults (Seidler et al., 2010; Song et al., 2014; King et al., 2018; Monteiro et al., 2019). This indicates a decreased segregation of the large-scale brain networks with advancing age (King et al., 2018; Cassady et al., 2019), which was significantly related to worse bimanual motor performance in older but not in younger adults (King et al., 2018; Monteiro et al., 2019).

One could argue that TMS mapping protocols are not sensitive enough to detect subtle changes in motor map organization. However, TMS-derived cortical motor representations acquired at rest have shown the ability to detect neuroplastic adaptations, e.g., in musicians (Pascual-Leone, 2001; Chieffo et al., 2016), after immobilization (Liepert et al., 1995) or following a transcutaneous electrical nerve stimulation (TENS) intervention (Meesen et al., 2011). Consequently, it could be postulated that the plasticity of cortical motor representations is mainly the result of training and/or learning and to a lesser extent the result of aging itself. In other words, if older adults keep performing daily manual activities at high intensity, their underlying sensorimotor representations may remain largely intact up to advanced ages.

It should be noted that aging might affect the brain’s motor network (Solesio-Jofre et al., 2014) more than isolated brain regions such as M1 alone. When using dual-site TMS to investigate projections from motor network nodes to M1, differences in connectivity in older as compared to younger adults have been identified (Rurak et al., 2021a,b; Verstraelen et al., 2021).

### Motor map distinctiveness

For the present work, two measures of cortical motor map distinctiveness of the FDI and ADM muscle representation where chosen, namely the CoG distance that describes the location of the two muscle representations relative to each other, and the cosine similarity in order to represent the 3-dimensional topographical congruence between the volume of the two muscle representations. As the general aging literature indicated reduced neural distinctiveness (i.e., dedifferentiation) in older as compared to younger adults (Sailer et al., 2000; Calautti et al., 2001; Mattay et al., 2002; Ward and Frackowiak, 2003; Koen and Rugg, 2019; Monteiro et al., 2020), we hypothesized a difference in the level of motor map distinctiveness with advancing age (i.e., a change in CoG distance and similarity of the cortical motor maps of different hand muscles). However, no aging effect on CoG distance or similarity of motor maps was found in the current sample.

Even though not explicitly investigated in the present work, there are two factors that possibly influence the relative localization and similarity of M1 muscle representations with advancing age. Firstly, structural CNS aging processes might immediately drive such changes. For example, gray matter atrophy in the M1 of older adults (Borzuola et al., 2020) could potentially lead to a decrease in cortical motor map overlap, and the decline in white matter integrity with advancing age (Barrick et al., 2010) potentially alters inter-regional connectivity. Secondly, changes in behavior with advancing age could in turn have an effect on the organization and functionality of M1. However, these factors are inseparably connected as structural changes in the CNS might in turn affect the behavior and vice versa.

As described for lateralization, resting-state fMRI evidence suggests a decrease in segregation of the large-scale brain networks with advancing age (Seidler et al., 2010; Song et al., 2014; King et al., 2018; Monteiro et al., 2019), suggesting age-related differences in the level of differentiation of the motor relative to other networks. Previous TMS studies investigating age-related changes in cortical motor maps at rest revealed contradictory findings. More specifically, while one study reported evidence for increased FDI representations in both hemispheres (Bernard and Seidler, 2012), other studies found no spatial alterations of the dominant FDI representation (Hehl et al., 2020) or even a decline in the extent of the dominant, but not non-dominant, APB representation (Coppi et al., 2014) with advancing age. For the ADM, a muscle less involved in dexterous hand movements, no changes of the motor representation were reported (Coppi et al., 2014; Hehl et al., 2020). However, the latter studies did not address the effects of aging on the relative CoG distance or similarity of different cortical motor maps.

Although we did not find changes in CoG distance and cosine similarity of hand muscle representations with advancing age, changes in motor map CoG location and overlap have been reported in the context of motor training and sensory interventions. More specifically, the long-term dexterous use of hands in, e.g., pianists as compared to non-musicians, has been associated with more distinct cortical motor representations of the APB, ADM, and extensor carpi radialis (ECR) muscles in the dominant hemisphere, but not with a change in CoG distance (Chieffo et al., 2016). Another study showed the effect of a 3-week sensory TENS intervention on the overlap and CoG location of the APB, ADM, ECR, and flexor carpi radialis (FCR) cortical motor maps. As opposed to the effect of long-term piano training, this TENS intervention was associated with an increase in the overlap between finger and forearm muscle representations, whereas again the CoG locations did not change (Meesen et al., 2011).

Therefore, it might be assumed that M1 is rather robust in the topography of its functional organization as measured by the CoG of cortical motor maps. Nonetheless, as mentioned before, a difference in overlap between different motor representations due to sensorimotor exposure has been reported (Meesen et al., 2011; Chieffo et al., 2016). Thus, the extent or excitability rather than the pure localization of motor maps might be influenced by behavior and/or aging, although the present work does not provide evidence for an aging effect on the level of distinctiveness of M1. Hence, when interpreting cortical motor maps as acquired with TMS, it must be kept in mind that these represent a complex interplay between the more stable anatomical/structural features and the more flexible/plastic functional organization of the motor cortex.

### Limitations

Firstly, hotspot and rMT were determined for the FDI and then used for the mapping of FDI and ADM, possibly resulting in a suboptimal targeting of the ADM (probably an underestimation, as the rMT of the ADM is likely slightly higher than the rMT of the FDI) as compared to targeting both muscles completely separately. However, this approach substantially reduces experimental duration and is common practice (Malcolm et al., 2006; Meesen et al., 2011; Raffin et al., 2015; Raffin and Siebner, 2019), especially when investigating more than one intrinsic hand muscle at the same time.

Secondly, the present study used a cross-sectional design, which is a common and highly feasible approach. Nonetheless, it poses a general limitation as systematic generation effects, such as environmental influences, cannot be excluded. Moreover, the chronological age of an individual (as measured in years lived since birth) seems less important for CNS and behavioral changes than the biological age [as measured in terms of, e.g., blood markers, physiological measures and signs of atrophy (Popescu et al., 2021) or metrics of structural and functional connectivity (Bonifazi et al., 2018) in brain imaging] (Schaefer, 2015) and possibly obscures aging effects, which might play an even bigger role in the current sample that was overall physically active (see Table 1). Although a longitudinal or accelerated longitudinal design would be most optimal to control for these effects, sufficient resources to carry out these challenging research designs are often not available. However, it should be noted that longitudinal studies are also inherent to limitations including for example restricted generalizability and dealing with missing data.

## Conclusion

In view of the general hypothesis of brain dedifferentiation, we investigated the effects of aging on different TMS-derived hand muscle representations in M1 that might be linked to changes in lateralization and/or distinctiveness. However, we could neither identify age-related differences in the lateralization of cortical motor representations of two intrinsic hand muscles, nor in their distinctiveness. This might imply that dedifferentiation mainly plays out at the level of overall brain organization, and more specifically, inter-network connectivity. However, when focusing on TMS-derived measures of lateralization and distinctiveness of the primary motor cortex, we found no clear evidence for this effect which may well be a consequence of daily performance of physical and/or manual activities that may support preservation of motor map representations.

## Data availability statement

The original contributions presented in this study are included in the article/supplementary material, further inquiries can be directed to the corresponding author.

## Ethics statement

The studies involving human participants were reviewed and approved by Ethische Commissie Onderzoek UZ/KU Leuven (EC Onderzoek). The patients/participants provided their written informed consent to participate in this study.

## Author contributions

MH, SS, and KC designed the study. MH and SVM performed the experiments. MH analyzed the data and wrote the manuscript with input from all authors. All authors contributed to the article and approved the submitted version.

## Abbreviations

ADM: abductor digiti minimi muscle
AIC: Akaike Information Criterion
APB: abductor pollicis brevis muscle
AREA: area of cortical motor representation
CNS: central nervous system
CoG: center of gravity
ECR: extensor carpi radialis muscle
EEG: electro-encephalography
EMG: electromyography
FCR: flexor carpi radialis muscle
FDI: first dorsal interosseus muscle
fMRI: functional magnetic resonance imaging
LQ: lateralization quotient
M1: primary motor cortex
MAXMEP: maximal motor evoked potential
MEP: motor evoked potential
MoCA: Montreal Cognitive Assessment
PPT: Purdue Pegboad Test (unimanual)
RMS: root mean square
rMT: resting motor threshold
SD: standard deviation
TENS: transcutaneous electrical nerve stimulation
TMS: transcranial magnetic stimulation
VOL: volume of cortical motor representation.
